# Dissecting the signaling pathways associated with the oncogenic activity of MLK3 P252H mutation

**DOI:** 10.1186/1471-2407-14-182

**Published:** 2014-03-14

**Authors:** Sérgia Velho, Ana Pinto, Danilo Licastro, Maria José Oliveira, Filipa Sousa, Elia Stupka, Raquel Seruca

**Affiliations:** 1 Instituto de Patologia e Imunologia Molecular da Universidade do Porto, Rua Dr. Roberto Frias, 4200-465 Porto,Portugal; 2 New Therapies Group, INEB-Institute for Biomedical Engineering, Porto, Portugal; 3 CBM S.c.r.l. AREA SCIENCE PARK, Trieste, Italy; 4 Center for Translational Genomics and Bioinformatics, San Raffaele Scientific Institute, Milan, Italy; 5 Medical Faculty of the University of Porto, Porto, Portugal

**Keywords:** Colorectal cancer, MLK3, WNT pathway, MSI, Planar cell polarity

## Abstract

**Background:**

MLK3 gene mutations were described to occur in about 20% of microsatellite unstable gastrointestinal cancers and to harbor oncogenic activity. In particular, mutation P252H, located in the kinase domain, was found to have a strong transforming potential, and to promote the growth of highly invasive tumors when subcutaneously injected in nude mice. Nevertheless, the molecular mechanism underlying the oncogenic activity of P252H mutant remained elusive.

**Methods:**

In this work, we performed Illumina Whole Genome arrays on three biological replicas of human HEK293 cells stably transfected with the wild-type MLK3, the P252H mutation and with the empty vector (Mock) in order to identify the putative signaling pathways associated with P252H mutation.

**Results:**

Our microarray results showed that mutant MLK3 deregulates several important colorectal cancer- associated signaling pathways such as WNT, MAPK, NOTCH, TGF-beta and p53, helping to narrow down the number of potential MLK3 targets responsible for its oncogenic effects. A more detailed analysis of the alterations affecting the WNT signaling pathway revealed a down-regulation of molecules involved in the canonical pathway, such as DVL2, LEF1, CCND1 and c-Myc, and an up-regulation of DKK, a well-known negative regulator of canonical WNT signaling, in MLK3 mutant cells. Additionally, FZD6 and FZD10 genes, known to act as negative regulators of the canonical WNT signaling cascade and as positive regulators of the planar cell polarity (PCP) pathway, a non-canonic WNT pathway, were found to be up-regulated in P252H cells.

**Conclusion:**

The results provide an overall view of the expression profile associated with mutant MLK3, and they support the functional role of mutant MLK3 by showing a deregulation of several signaling pathways known to play important roles in the development and progression of colorectal cancer. The results also suggest that mutant MLK3 may be a novel modulator of WNT signaling, and pinpoint the activation of PCP pathway as a possible mechanism underlying the invasive potential of MLK3 mutant cells.

## Background

Mixed-lineage kinase 3 (MLK3) belongs to a family of seven different mammalian MLKs, that are clustered in three subgroups accordingly with their structural similarities: the MLKs (MLK1, MLK2, MLK3, MLK4); the dual-leucine-zipper-bearing kinases (Dlk and Lzk); and zipper sterile-α-motif kinase (Zakα and Zakβ) [1].

MLK3 protein is composed by a Src-homology-3 (SH3) domain, located at the amino terminus of the protein, followed by a kinase domain, a leucine-zipper region, a Cdc42/RAC interacting binding motif (CRIB) and a Proline/Serine/Threonine-rich (P/S/T-rich) carboxy terminal domain [1-4]. All these domains show a very high degree of homology between MLK members (MLK1-MLK4) [1], except the carboxy-terminus P/S/T- rich domain which is the less conserved region among MLK proteins [1].

MLK3 is a serine/threonine protein kinase that regulates the mitogen-activated protein kinase (MAPK) pathway, activating ERK, p38 and JNK in response to extracellular signals [1,5]. Additionally, functional studies have demonstrated that overexpression of wild-type MLK3 leads to morphological transformation of NIH 3T3 fibroblasts and growth in soft agar, a capacity that is MEK/ERK dependent [6]. Further, MLK3 has been demonstrated to function as a scaffolding protein, involved in the formation of a multiprotein complex containing MLK3/BRAF/RAF1 [1,5,7,8]. The formation of this complex was shown to be important for the activation of wild-type BRAF and, consequently, to the activation of ERK signaling [1,5,7,8]. Furthermore, MLK3 was reported to be important for the proliferation of tumor cells, bearing either oncogenic KRAS or neurofibromatosis-1 (NF1) or NF2 inactivating mutations [8]. In addition, the MLK3 signaling activation was associated with an increase in the migratory and invasive capacity of tumor cells in gastric [9], breast [10-12], lung [13] and ovarian [14] cancers. All together, these observations implicate MLK3 as a cancer-related gene although, until recently, nothing was known about MLK3 gene deregulation in primary cancer tissues.

Our group reported the occurrence of MLK3 mutations in microsatellite unstable (MSI) gastrointestinal tumors (both sporadic and hereditary forms) in a frequency of about 20%. Using *in vitro* transforming assays, we demonstrated that several MLK3 mutations affecting different domains of the protein had transforming potential when compared to cells expressing the wild-type and the kinase-dead forms of the protein [15]. These results were further supported by *in vivo* studies in which one of the two most transforming mutations (P252H – located in the kinase domain) was found to be tumorigenic and to give rise to highly invasive tumors when subcutaneously injected in nude mice. Thus, our previous work pointed mutant MLK3 as a new oncogene in MSI gastrointestinal cancers, however, the signaling pathways associated to its oncogenic activity remained to be explored.

In this work, we aimed at identifying the signaling pathways associated to mutant MLK3, in particular the P252H mutation. The reasons underlying the choice for this mutation were: (a) it was one of the most transforming mutations previously analyzed; (b) showed high tumorigenic capacity with strong invasive potential in *in vivo* studies; and (c) it was located in the kinase domain which is an important domain for the regulation of downstream signaling pathways.

The results showed that P252H mutation interferes with important colorectal cancer-associated signaling pathways such as WNT, MAPK, NOTCH, TGF-β and P53.

## Methods

### cDNA constructs and mutagenesis

Wild-type MLK3 and mutant sequences were cloned into pLENTID6/V5 directional TOPO (Invitrogen). Mutant MLK3 P252H sequence was generated by site-directed mutagenesis using the MLK3 wild-type sequence cloned in pLENTID6/V5 as template. pLENTID6/V5 empty vector (Mock) was obtained by the insertion of a small fragment of cDNA in order to circularize the plasmid.

### Cell lines

Human HEK293 were maintained in DMEM (Gibco, Invitrogen) supplemented with 10% FBS and 1% penicillin–streptomycin (Gibco, Invitrogen), and were incubated in a humidified chamber with 5% CO_2_ at 37°C.

### Transfections

For HEK293 stable transfections, ViraPower Lentiviral Expression kit (Invitrogen) was used for the transduction of the MLK3 wild-type and mutant P252H sequences as well as the empty vector. Lentiviral transduction was performed following the manufacturer’s instructions. Transduced cells were selected by antibiotic resistance (blasticidin, 12 μg/ml) (Gibco, Invitrogen). The expression levels of MLK3 in the different clones selected were measured by western-blot.

### RNA extraction and cDNA synthesis

Total RNA was isolated from cell lines using TriPure Isolation Reagent (Roche Applied Science), following manufacturer’s instructions. Complementary DNA was synthesized from 1 μg of total RNA using SuperScriptII Reverse Transcriptase (Invitrogen) and Random Primers (Invitrogen).

### Labeling and hybridization

Five hundred ng aliquots of RNA from the samples were quality checked using the Agilent 2100 Byoanalyzer and only samples above integrity quality number (RIN) 8 [16] were used and amplified according to the specifications of the Illumina® TotalPrep™ RNA Amplification Kit (Ambion, Austin, TX, USA). The cRNA samples were applied to the arrays of Sentrix® Human-v6 Expression BeadChip (Illumina, San Diego, CA, USA) and hybridized according to manufacturer's specifications. The Sentrix BeadChips were scanned with the Illumina's Beadarray system 500G Scanner (Illumina®).

### Microarray data analysis

The signal intensity was extracted from the hybridization images, background subtracted and normalized using Illumina Inc. BeadStudio software version 3.3.7. The data produced was checked against the Illumina internal quality controls and loaded into the Bioconductor software [17,18]. To identify differentially expressed genes based on a moderate t-test, the bioconductor Limma package [19] was used. Genes were selected based on a *p*-value cut-off (after adjustment) of *p* < 0.01 to control the false discovery rate (FDR) [20]. To test the association of selected differentially expressed genes with KEGG pathways [21], information provided in the Illumina annotation files (Sentrix® Human-v6 Expression BeadChip) Hypergeometric test available in the GOstats packages [22] was used. A *p*-value cut off of 0.05 was considered. Microarray data can be found at the GEO repository with the accession number GSE54611.

### Real-time PCR

One μl of cDNA was added to 10 μl Real-Time PCR mixtures containing 1x TaqMan® Universal PCR Master Mix, No AmpErase® UNG (Applied Biosystems) and 1x TaqMan® MGB specific probes and primers mix. Taqman expression assays for BMP6 (Hs01099594_m1), CCND1 (Hs00277039_m1), FZD10 (Hs00273077_s1), LEF1 (Hs00212390_m1) were purchased from Applied Biosystems. The eukaryotic 18S rRNA assay (Hs99999901_s1; Applied Biosystems) was used as an endogenous control gene. Standard TaqMan thermocycling conditions were used: 1 cycle of 2 minutes at 50°C, 1 cycle of 10 minutes at 95°C, 40 cycles of 15 seconds at 95°C followed by 1 minute at 60°C in an ABI Prism 7000 (Applied Biosystyems). Real-Time PCR assays (absolute quantification) were performed in, at least, three biological replicas.

### Statistical analyses

For statistical analyzes of *in vitro* transformation assays a *t*-student test was used and *p* < 0.05 was taken as statistically significant. Specific statistical tests used for microarray interpretation are embedded in the corresponding materials and methods section.

## Results and discussion

### MLK3 P252H mutation affects fundamental colorectal cancer-associated pathways

**Figure 1:**
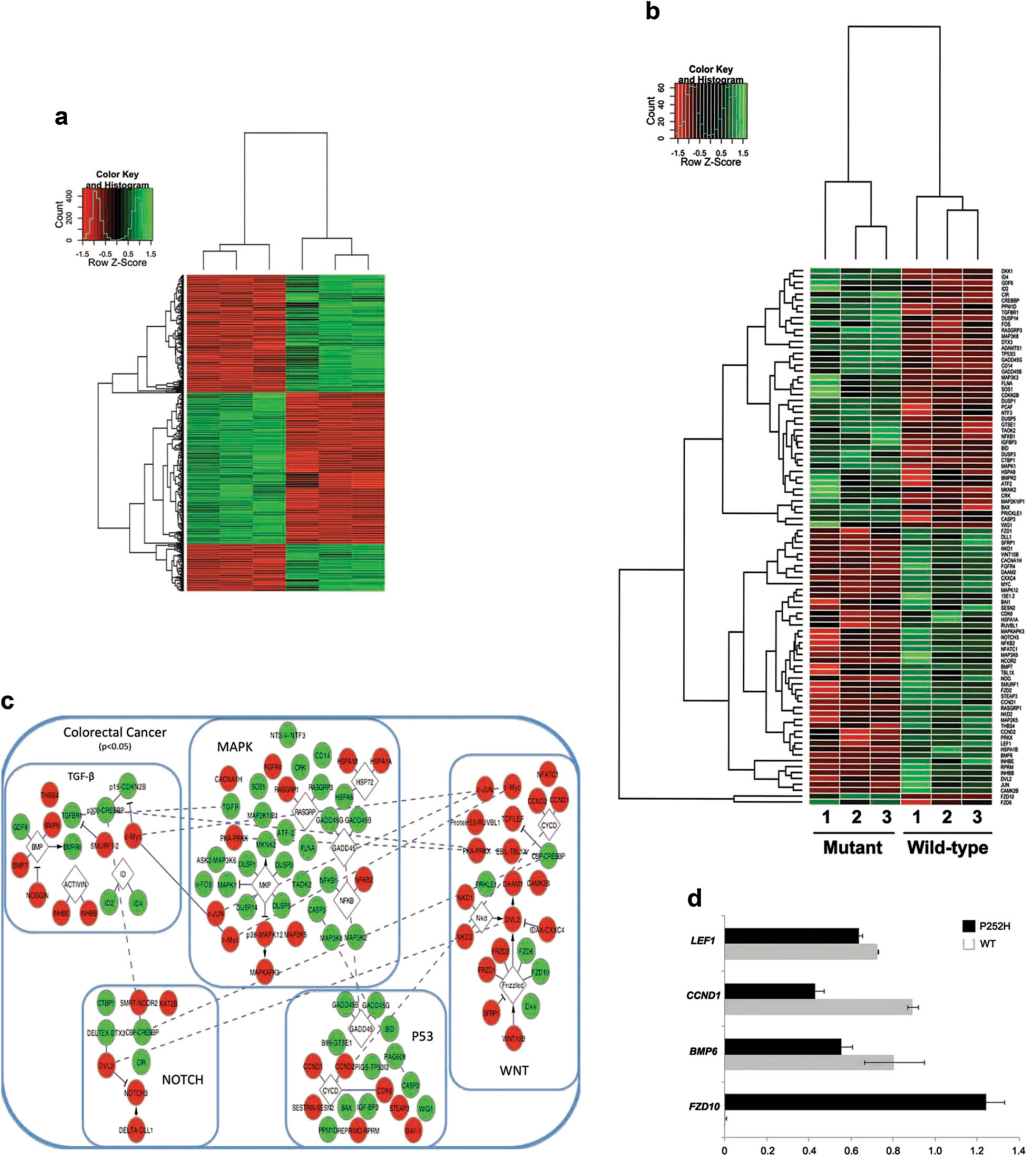
**Expression profiling of biological triplicates of HEK293 cells transfected with mutant and wild-type MLK3. (a)** Heatmap of the 445 genes which clearly classify the mutant vs the wild-type, selected on the basis of a 2 log-fold change in expression between cells transfected with P252H mutant and wild-type forms of MLK3, and eliminating genes which were differentially expressed between the Mock and wild-type transfection (red=down-regulated in P252H mutant MLK3, green=up-regulated in P252H mutant MLK3). **(b)** Illustrates a subset of the heatmap, focusing on genes which are part of the colorectal cancer pathway which is significantly affected (*p*-value = 0.03). **(c)** Graphical representation of the colorectal pathway (larger rounded rectangle) and relevant pathways contained within it (smaller rounded rectangles), such as MAPK, WNT, TGF-β, NOTCH and p53, indicating differentially expressed genes (ovals) present in Figure 1, their relationships to each other (solid line indicates a direct protein-protein interaction, T shaped ending for inhibition interactions, arrow ending for activation interactions, and no ending for other known interactions), as well as the direction of the expression change (red = down-regulated in P252H mutant MLK3 transfection, green = up-regulated in mutant MLK3). White Diamond shaped boxes indicate entire gene families, which are significantly affected, genes within the families are shown as colored ovals. Long dashed lines indicate genes, which are present in multiple pathways. **(d)** Graphical representation of Real-time PCR quantification of mutant vs wild-type MLK3 targets (LEF1, CCND1, BMP6 and FZD10) selected from expression microarrays.

In order to assess the effect of a tumorigenic MLK3 mutant on a genome-wide level, we performed microarray-based expression profiling experiments. We used Illumina Whole Genome arrays on three biological replicas of human HEK293 cells stably transfected with the wild-type MLK3, with the MLK3 P252H mutation and with the empty vector (Mock). The expression profiles were obtained by comparing all biological replicas from each transfection experiment. Colorectal cancer cell lines were not used in this experiment, as proteins from the MAPK pathway are frequent mutation targets in this type of cancer, and would most likely interfere with the interpretation of the results. The expression profiles were compared to identify genes that were differentially expressed at least 2 log-fold (FDR < 0.01) between wild-type and Mock, as well as between P252H and Mock. A final set of 445 genes was identified which showed significant differential expression only between P252H and wild-type and not between wild-type and Mock (Figure 1a). The most statistically significantly differentially expressed genes are displayed in Figure 1b. The genes identified were significantly enriched (*p* < 0.05) in several KEGG pathways (Table 1) involved in overall biosynthesis processes, as well as in response in disease relevant processes. Interestingly, the colorectal pathway, which encompasses several relevant pathways such as WNT, MAPK, NOTCH, TGF-β and P53, was significantly over-represented (Figure 1c). These signaling pathways are crucial to maintain intestinal epithelium homeostasis by balancing the rate of proliferation, apoptosis, and differentiation along the crypt-villus axis, and their de-regulation is commonly associated to colorectal cancer initiation and progression [23]. Corroborating our results, a recent study using both *in vivo* and *in vitro* approaches showed that MLK3 signaling is important in intestinal mucosal healing and epithelial cell motility [24], therefore implicating MLK3 signaling in the maintenance of intestinal epithelial homeostasis.

**Table 1:** KEGG Pathways significantly affected by mutant MLK3

**KEGG**	***p-value***	**Odds**	**Exp count**	**Count**	**Size**	**Term**
**ID**		**Ratio**				
970	0.000	4.669	5	16	35	Aminoacyl-tRNA biosynthesis
290	0.001	7.682	2	7	12	Valine, leucine and isoleucine biosynthesis
5120	0.001	2.477	11	21	68	Epithelial cell signaling in Helicobacter pylori infection
251	0.004	3.184	5	11	30	Glutamate metabolism
630	0.009	4.693	2	6	13	Glyoxylate and dicarboxylate metabolism
4115	0.013	1.984	11	18	68	p53 signaling pathway
330	0.014	2.517	5	11	35	Arginine and proline metabolism
3020	0.018	2.923	4	8	23	RNA polymerase
5020	0.026	2.948	3	7	20	Parkinson's disease
670	0.027	3.283	2	6	16	One carbon pool by folate
5210	0.03	1.721	13	20	84	Colorectal cancer
600	0.031	2.155	6	11	39	Sphingolipid metabolism
260	0.038	1.995	7	12	45	Glycine, serine and threonine metabolism
5040	0.041	2.241	5	9	31	Huntington's disease
5110	0.044	2.01	6	11	41	Cholera - infection

In order to further validate the microarray data, a set of differentially expressed genes (LEF1, CCND1, FZD10, and BMP6) were selected for validation by real-time PCR in HEK293 cells stably expressing MLK3 wild-type or MLK3 P252H. The results obtained with the microarrays were validated for all genes tested (Figure 1d).

Of particular interest are the alterations that mutant MLK3 induces in the WNT pathway. Activation of canonical WNT signaling through WNT/β-catenin cascade has traditionally been regarded as a critical player in colorectal tumorigenesis [25]. More recently, accumulating evidence supports a role for the non-canonical WNT planar cell polarity (PCP) pathway, a signaling cascade involved in the polarization of cells during tissue remodeling, and cell adhesion and motility, in cancer progression, invasion, metastasis, and angiogenesis [26-28]. A more detailed analyzes of our microarray data showed that the expression of several molecular components of the canonical pathway, such as DVL2, LEF1, CCND1 and c-MYC were down-regulated in MLK3 mutant cells, and the expression of DKK, a well-known negative regulator of canonical WNT signaling [29], was up-regulated (Figure 1c and d). On the other hand, genes encoding two WNT receptors known to act as negative regulators of the canonical WNT/β-catenin signaling cascade and as positive regulators of the PCP pathway, FZD6 and FZD10, were found to be up-regulated in P252H cells (Figure 1c and d). Taken together, the down-regulation of DVL2, LEF1, CCND1 and c-MYC, and the up-regulation of DKK and FZD receptors suggest a role of mutant MLK3 as a molecular switch between canonic and non-canonic WNT signaling. In accordance, it was recently reported that MLK3 reduces the expression of β-catenin/TCF downstream targets by promoting the interaction between β-catenin and KLF4, a known repressor of β-catenin/TCF transcriptional activity [30]. Furthermore, in accordance with a role of PCP in colorectal cancer, FZD10 was recently demonstrated to be up-regulated in colorectal cancers and matched liver metastases, and its over-expression was associated with the activation of non-canonical WNT pathway [31,32].

## Conclusion

In conclusion, our results provide an overall view of the expression profile associated with mutant MLK3, and they support the functional role of mutant MLK3 by showing a deregulation of several signaling pathways known to play important roles in the development and progression of colorectal cancer. The results also suggest that mutant MLK3 may be a novel modulator of WNT signaling, and pinpoint the activation of the PCP pathway as a possible mechanism underlying the invasive potential of MLK3 mutant cells. Nevertheless, further studies are required in order to validate this hypothesis in a panel of gastrointestinal cell lines and human primary tumors, to determine if the altered signaling pathways are common to other MLK3 mutations, and to investigate the role of mutant MLK3 in the context of mutant KRAS and BRAF genes.

## Abbreviations

MLK: Mixed lineage kinase; WNT: Wingless type; BMP: Bone morphogenetic protein; TGF-β: Transforming growth factor β; MSI: Microsatellite instability; PCP: Planar cell polarity; DKK: Dickkopf; FZD: Frizelled; DVL2: Dishevelled; LEF1: Lymphoid enhancer binding factor 1; CCND1: Cyclin D1; KLF4: Krupfel-like factor; c-MYC: v-myc avian myelocytomatosis viral oncogene homolog; TCF: T cell-specific transcription factor; PCR: Polymerase chain reaction; MEK: MAP kinase-ERK kinase; ERK: Extracellular signal-regulated kinase; MAPK: Mitogen-activated protein kinase; JNK: c-Jun amino-terminal kinase; BRAF: v-raf murine sarcoma viral oncogene homolog B; KRAS: Kirsten rat sarcoma viral oncogene homolog; Dlk: Dual-leucine-zipper-bearing kinase; Lzk: Leucine-zipper-bearing kinase; Zak: Zipper sterile-α-motif kinase; SH3: Src-homology-3.

## Competing interests

The authors declare that they have no competing interests.

## Authors’ contributions

SV contributed to the acquisition, analysis and interpretation of data and drafted the manuscript; AP participated in the acquisition, analysis and interpretation of data; DL contributed to the acquisition, analysis and interpretation of data; MJO was responsible for the conception and design of the experimental system and critically reviewed the manuscript; FS was involved in the acquisition, analysis and interpretation of data; ES contributed for the conception and design of the experimental system and critically reviewed the manuscript; RS contributed to the conception and design of the project and experimental system, interpretation of data, and was responsible for the final approval of the version to be published.

## Pre-publication history

The pre-publication history for this paper can be accessed here:

http://www.biomedcentral.com/1471-2407/14/182/prepub
